# Antihyperglycemic and Antioxidant Effects of *Salacia reticulata* and *Caralluma tuberculata* in Alloxan-Induced Diabetic Female Rats

**DOI:** 10.3390/pharmaceutics18070785

**Published:** 2026-06-26

**Authors:** Naglaa Gamil Shehab, Rania H. Shalaby, Shabana Anjum, Surendra Singh Rawat, Eslam Mahmoud Alrefaee, Fatimah Saad Altamimi, Hanaa Al-Shafea, Naiba Khusrau, Stefan S. Du Plessis, Temidayo S. Omolaoye

**Affiliations:** 1 Pharmaceutical Sciences Department, College of Pharmacy, Dubai Medical University, Dubai P.O. Box 20170, United Arab Emirates; 2 Pharmacognosy Department, Faculty of Pharmacy, Cairo University, Cairo 11562, Egypt; 3 Biomedical Sciences Department, College of Medicine, Dubai Medical University, Dubai P.O. Box 20170, United Arab Emirates; dr.rania@dmu.ae; 4 Pharmacology Department, Faculty of Medicine, Tanta University, Tanta 31527, Egypt; 5 College of Medicine, Mohammed Bin Rashid University of Medicine and Health Sciences, Dubai Health, Dubai P.O. Box 505055, United Arab Emirates; shabana.anjum@dubaihealth.ae (S.A.); stefan.duplessis@dubaihealth.ae (S.S.D.P.); 6 Research and Graduate Studies, Mohammed Bin Rashid University of Medicine and Health Sciences, Dubai Health, Dubai P.O. Box 505055, United Arab Emirates; surendrasingh.rawat@dubaihealth.ae; 7 College of Pharmacy, Dubai Medical University, Dubai P.O. Box 20170, United Arab Emirates; ema20210205@dpc.edu (E.M.A.); fss20220241@dpc.edu (F.S.A.); had20230270@dpc.edu (H.A.-S.); nak20210221@dpc.edu (N.K.)

**Keywords:** diabetes mellitus, alloxan, *Salacia reticulata*, *Caralluma tuberculata*, oxidative stress, UPLC–MS/MS

## Abstract

**Objective**: Diabetes mellitus (DM) is a major metabolic disorder associated with hyper-glycemia and oxidative stress. Traditional medicinal plants remain important sources of bioactive compounds with potential antidiabetic activity. *Salacia reticulata* and *Caralluma tuberculata* are two important medicinal plants that have been reported to have antidiabetic effects. The growing burden of type 2 diabetes and the need for therapies that address both hyperglycemia and oxidative stress underscore the necessity to investigate these two medicinal plants. Therefore, the current study evaluated the antihyperglycemic, antioxidant, and protective effects of *Salacia reticulata* and *Caralluma tuberculata* in an alloxan-induced diabetic female rat model. **Methods**: Ethanolic extracts of *S. reticulata* and *C. tuberculata* were characterized by total phenolic content (TPC), total flavonoid content (TFC), DPPH radical-scavenging assay, and UPLC–MS/MS metabolite profiling. Female Wistar rats (n = 42) were randomly assigned to seven groups (n = 6/group), including normal control, diabetic control, extract-treated non-diabetic groups, diabetic extract-treated groups, and a metformin-treated diabetic group. Diabetes was induced by alloxan (130 mg/kg), followed by oral treatment for 8 days with extracts or metformin (500 mg/kg/day). Fasting blood glucose, oral glucose tolerance, serum malondialdehyde (MDA), antioxidant markers (SOD1, GSH, and CAT), and liver and kidney histopathology were assessed. **Results**: Both plant extracts significantly reduced fasting blood glucose compared with baseline, with *S. reticulata* showing a greater reduction (22.8%) than *C. tuberculata* (12.3%), and a response comparable to metformin (27.4%). Diabetic rats exhibited increased MDA and reduced antioxidant enzyme activities. *C. tuberculata* significantly lowered MDA levels and increased SOD1 activity, suggesting moderate antioxidant effects, whereas *S. reticulata* showed higher phenolic and flavonoid contents and the highest DPPH scavenging activity. UPLC–MS/MS identified 33 compounds in *S. reticulata* and 24 in *C. tuberculata*. Histopathological findings supported improvement of diabetes-associated renal and hepatic damage. **Conclusions**: Within the eight-day experimental period, both extracts demonstrated significant acute antidiabetic and antioxidant effects with distinct redox–metabolic profiles. However, further long-term studies are recommended to evaluate their sustained efficacy, safety, and potential as complementary therapeutic agents for diabetes management.

## 1. Introduction

Diabetes mellitus (DM) is a chronic metabolic disorder characterized by persistent hyperglycemia resulting from defects in insulin secretion and insulin action, or both. The International Diabetes Federation reports that 589 million adults worldwide are affected by diabetes mellitus, and the estimated number of adults is projected to reach 853 million by 2050 [1], with 90% of people living in low- and middle-income countries, highlighting the increasing health burden [2]. Metformin has long been used as a first-line treatment for type 2 diabetes mellitus (T2DM). The treatment strategies for T2DM have changed in recent years. According to the American Diabetes Association, new medicines such as GLP-1 receptor agonists and GIP receptor agonists are central components for managing both diabetes and its related comorbidities, such as cardiovascular disease, obesity, and liver disease [3].

Metformin’s primary mechanisms include suppression of hepatic glucose production and improvement of insulin sensitivity. Although generally well tolerated, metformin may cause gastrointestinal disturbances, vitamin B12 deficiency with prolonged use, and, in rare cases, lactic acidosis, particularly in patients with renal impairment or comorbid conditions [4,5,6]. These limitations have encouraged the continued search for complementary therapeutic options [7]. Traditional medicinal plants have long been used to manage diabetes. The World Health Organization (WHO) recognizes traditional medicine as an important component of primary healthcare in many regions [8]. Given the limited access to conventional medical care, plant-based therapies remain widely used. Scientific validation of these medicinal plants is therefore essential to substantiate their efficacy and safety [9].

*Salacia reticulata* (*S. reticulata*), a climbing woody plant belonging to the Celastraceae family and distributed across tropical regions of India and Sri Lanka, has been extensively studied for its antidiabetic properties [7,10,11]. Phytochemical investigations have identified mangiferin, kotalanol, salacinol, glycosides, coumarins, carbohydrates, and phytosterols within the plant [10,12]. These constituents are reported to inhibit intestinal α-glucosidases, thereby reducing postprandial glucose levels [13]. In addition, *S. reticulata* has been reported to have antioxidant, lipid-lowering, and hepatoprotective activities, underscoring its relevance in the management of metabolic disorders [14].

Similarly, *Caralluma tuberculata* (*C. tuberculata*), a succulent perennial herb of the Apocynaceae family, native to South Asia and parts of the Middle East, contains diverse bioactive compounds including pregnane glycosides, flavone glycosides, saponins, triterpenes, and other flavonoids [15,16]. Traditionally, it has been used for weight management, rheumatism, and glycemic control [17], suggesting potential metabolic regulatory effects. The growing burden of type 2 diabetes and the need for therapies that address both hyperglycemia and oxidative stress underscore the necessity to evaluate these two medicinal plants. Although individual studies have examined their antidiabetic properties, a direct comparison of their antihyperglycemic and antioxidant effects within a single experimental framework remains limited. Although alloxan-induced diabetes may show selected type 2-like metabolic features when β-cell destruction is partial, its primary mechanism is direct β-cell cytotoxicity; therefore, the present model is best described as an alloxan-induced diabetic model rather than a definitive type 2 diabetes model [18,19]. The present study is the first to directly compare the antihyperglycemic and antioxidant effects of *S. reticulata* and *C. tuberculata* using the same alloxan-induced diabetic rat model, treatment dose, duration, and analytical methods, revealing distinct yet complementary mechanisms of action. Therefore, this study aimed to evaluate and compare the antihyperglycemic activity, modulation of oxidative stress, and antioxidant potential of *S. reticulata* and *C. tuberculata* in an alloxan-induced experimental diabetic rat model, using metformin as a reference standard. In addition to biochemical assessments, histopathological examination of hepatic and renal tissues was conducted to evaluate structural alterations associated with diabetes and treatment response. Phytochemical characterization, including quantification of total phenolic content (TPC) and total flavonoid content (TFC), and Liquid Chromatography–Mass Spectrometry (LC-MS) profiling, was performed to relate biological effects to the constituent composition.

## 2. Materials and Methods

### 2.1. Chemicals and Reagents

Analytical-grade solvents and reagents were used throughout the study. Absolute ethanol was obtained from Wunstorfer Strasse, Hannover, Germany. Methanol (HPLC grade) and sodium hydroxide were purchased from Fisher Scientific, Loughborough, UK. Folin–Ciocalteu reagent, aluminum chloride (AlCl_3_), sodium nitrite (NaNO_2_), sodium carbonate (Na_2_CO_3_), sodium hydroxide (NaOH), alloxan monohydrate (≥98%), acetonitrile, ammonium formate (≥98%), and 2,2-diphenyl-1-picrylhydrazyl (DPPH) were purchased from Sigma-Aldrich, St. Louis, MO, USA. Glucophage XR 500 mg tablets (metformin hydrochloride; Merck, Darmstadt, Germany) were obtained from a local pharmacy (Merck Healthcare, Dubai, United Arab Emirates).

### 2.2. Plant Material and Standardization

*S. reticulata* powder root was sourced from India (Sun Agri Export, Chennai, India; Batch No. SAR-2024-09), and *C. tuberculata* fleshy stems were obtained from a greenhouse in Warsan, Dubai, United Arab Emirates (September 2024; Batch No. CT-GH-2024-09). Both plants were taxonomically verified by Prof. Naglaa Gamil Shehab (Pharmaceutical Sciences Department, Dubai Medical University). Voucher specimens were kept at the Herbarium of the Pharmaceutical Sciences Department (#28-09-24). Raw materials were standardized prior to extraction and microscopically investigated. The extracts were characterized by measuring total phenolic and flavonoid contents (TPC and TFC respectively) as described below. Batch-to-batch consistency was confirmed by the antioxidant activity using DPPH assay.

#### Extraction Procedure

Eight hundred grams from each plant under investigation was used. *Caralluma tuberculata* succulent stems were cut into small pieces. Both plants were macerated in absolute ethanol (1150 mL ×2) at room temperature using a sonicator for one week. The sonicator temperature was checked regularly during sonication, and whenever a temperature rise was observed, sonication was paused, and cooling was applied until the sample returned to room temperature. The extracts were filtered and concentrated under reduced pressure at 50 °C using a rotary evaporator (Buchi, Gallen, Switzerland). Dried extracts were stored at 4 °C in amber airtight vials under a nitrogen atmosphere to minimize oxidative degradation. Storage stability was confirmed by repeated DPPH assay at 4-week intervals. All biological and analytical experiments were conducted within 8 weeks of extraction.

### 2.3. Phytochemical Analyses

#### 2.3.1. Total Phenolic Content (TPC)

The TPC of each plant extract was determined using the Folin–Ciocalteu colorimetric method with gallic acid, as described by Singleton and Rossi and further modified by Naglaa et al. [20]. Briefly, 1 mL of extract was mixed with 1 mL of Folin–Ciocalteu reagent, diluted with distilled water, and incubated with 7% sodium carbonate (Na_2_CO_3_) at room temperature. After 90 min, absorbance was measured at 750 nm using a UV–Vis spectrophotometer (UV-1800) (Shimadzu Corporation, Kyoto, Japan). Gallic acid standard solutions (10–100 µg/mL) were prepared and processed using the same Folin–Ciocalteu procedure. A calibration curve was generated by plotting absorbance against gallic acid concentration, and total phenolic content was expressed as mg gallic acid equivalents (GAEs) per gram of dry extract. Results were expressed as milligrams of gallic acid equivalents per gram of dry extract.

#### 2.3.2. Total Flavonoid Content (TFC)

The TFC was quantified using the aluminum chloride colorimetric assay with quercetin as the standard [21]. Total flavonoid content was quantified using the aluminum chloride colorimetric assay with quercetin as the standard. Total flavonoid content was determined using the aluminum chloride colorimetric method. Briefly, the extract was mixed with distilled water and 5% sodium nitrite and incubated for 5 min. Thereafter, 10% of aluminum chloride was added and incubated for another 5 min. Subsequently, 1 M of sodium hydroxide was added to stop the reaction. Absorbance was measured at 510 nm using a UV–Vis spectrophotometer (UV-1800). Quercetin standard solutions (10–100 µg/mL) were prepared and treated under identical conditions to generate a calibration curve, and total flavonoid content was expressed as mg quercetin equivalents (QEs) per gram of dry extract. Results were expressed as milligrams of quercetin equivalents per gram of dry extract.

#### 2.3.3. UPLC–MS/MS Analysis

Metabolite profiling of the plant extract was performed using reverse-phase ultra-performance liquid chromatography coupled with tandem mass spectrometry (UPLC–MS/MS) (Shimadzu Corporation, Kyoto, Japan). Sample preparation, chromatographic separation, mass spectrometric acquisition, and data processing were carried out as described below.

##### Sample Preparation

For UPLC–MS/MS analysis, 50 mg of the dried sample was dissolved in 1 mL of reconstitution solvent consisting of water: methanol: acetonitrile at a ratio of 50:25:25, *v*/*v*/*v*. The mixture was vortexed for 2 min, followed by ultrasonication for 10 min to ensure complete extraction of metabolites. The sample was then centrifuged at 10,000 rpm for 10 min, and the clear supernatant was collected.

An aliquot of 50 µL of the stock solution was diluted to a final volume of 1000 µL using the same reconstitution solvent, giving a final injected concentration of 2.5 µg/µL. A volume of 10 µL was injected for UPLC–MS/MS analysis. The reconstitution solvent was also injected under the same conditions as a blank sample to monitor background signals and solvent-derived features.

##### Chromatographic and Mass Spectrometric Conditions

The bioactive constituents of the plant extract were analyzed using a Sciex ExionLC system (SCIEX, Framingham, MA, USA) coupled to a TripleTOF 5600+ mass spectrometer (SCIEX, Framingham, MA, USA). Chromatographic separation was achieved on a Waters XSelect HSS (Waters Corporation, Milford, MA, USA) T3 C18 column, 100 Å, 2.5 µm, 2.1 × 150 mm, equipped with a Phenomenex (Phenomenex, Torrance, CA, USA), in-line precolumn filter, 0.5 µm × 3.0 mm. The column temperature was maintained at 40 °C, the flow rate was set at 0.3 mL/min, and the injection volume was 10 µL.

The mobile phase consisted of aqueous ammonium formate buffer and acetonitrile as the organic phase. For the ESI-positive mode, the aqueous phase was 5 mM ammonium formate buffer, pH 3, containing 1% methanol and 0.01% formic acid. For the ESI-negative mode, the aqueous phase was 5 mM ammonium formate buffer, pH 8, containing 1% methanol. Gradient elution was programmed as follows: 0–1 min, 95% aqueous phase and 5% acetonitrile; linear ramp to 5% aqueous phase and 95% acetonitrile by 21 min; re-equilibration at 95% aqueous phase and 5% acetonitrile at 28.0 min; followed by a high-organic wash at 5% aqueous phase and 95% acetonitrile from 28.1 to 35 min.

Mass spectrometric detection was performed in high-resolution TOF-MS mode with information-dependent acquisition MS/MS, IDA-MS/MS, over an *m*/*z* range of 50–1000 in both positive and negative ionization modes. The source parameters were set as follows: nebulizer gas, GS1, 45; auxiliary gas, GS2, 45; curtain gas, CUR, 25; source temperature, 500 °C; and ion spray voltage, +4500 V for positive ion mode and −4500 V for negative ion mode.

The IDA parameters included a declustering potential of 80 V, collision energy of ±35 V, collision energy spread of 20 V in positive mode and 15 V in negative mode, 15 candidate ions per cycle, 10 ppm mass tolerance, dynamic background subtraction, and a cycle time of 0.65 s. Detailed LC acquisition parameters are provided in the Supplementary Materials.

##### Calibration and Mass Accuracy

External calibration was performed using mixed standard solutions of representative compounds, including quercetin, luteolin, mangiferin, and caffeic acid, prepared at six concentration levels ranging from 0.1 to 50 µg/mL. Calibration curves showed good linearity, with correlation coefficients of R^2^ ≥ 0.998. Mass accuracy was verified before each analytical batch using lock-mass calibration with leucine-enkephalin, [M + H]^+^ = 556.2771.

##### Data Acquisition and Processing

Data acquisition was carried out using Analyst TF 1.7.1, while preliminary visualization and compound inspection were performed using PeakView 2.2 and MasterView 1.1. Further data processing and metabolite annotation were conducted using MS-DIAL 4.9.

MS-DIAL processing was performed using MS1/MS2 tolerances of 0.05 Da, a mass slice width of 0.05 Da, a minimum peak height threshold of 1000 a.u., smoothing level of 2 scans, and an identification score threshold of ≥0.7. The databases used for metabolite annotation included GNPS negative mode, 2351 records; GNPS positive mode, 8782 records; ReSpect positive mode, 2737 records; and ReSpect negative mode, 1573 records. Each database was processed separately. Detailed MS-DIAL analysis parameters are provided in the Supplementary Materials.

##### Feature Extraction and Metabolite Annotation

Feature extraction was performed from the total ion chromatogram, TIC, according to the following criteria: signal-to-noise ratio greater than 10, mass error within ±10 ppm, and sample-to-blank intensity ratio greater than 3. These criteria were applied to reduce background-derived signals and improve the reliability of non-targeted metabolite detection.

Putative compounds were annotated by comparing retention times, precursor ions, product ions, accurate mass values, and MS/MS fragmentation patterns with authentic reference standards when available, as well as with published spectral data and spectral libraries. Quantification was performed using calibration curves constructed from authentic standards when available [22,23].

#### 2.3.4. Determination of Antioxidant Activity (DPPH Assay)

Antioxidant capacity was evaluated using the DPPH radical scavenging method. The antioxidant activity of the plant extracts was evaluated using the 2,2-diphenyl-1-picrylhydrazyl (DPPH) radical scavenging assay, as previously described by Shehab et al. [20]. Briefly, 0.3 g of each dried extract was dissolved separately in 20 mL of absolute ethanol and sonicated until complete dissolution. For each extract, three sets of test tubes were prepared: sample tubes (n = 3) containing 1 mL of stock solution and 1 mL of ethanol; DPPH-treated tubes (n = 3) containing 1 mL of stock solution, 1 mL of ethanol, and 1 mL of DPPH solution; and control tubes (n = 3) containing 2 mL of ethanol and 1 mL of DPPH solution without extract.

All tubes were incubated in the dark at room temperature for 30 min to prevent DPPH photodegradation. Absorbance was measured at 517 nm using a UV–Vis spectrophotometer (UV-1800). The percentage inhibition of DPPH radicals was calculated using the following equation:

% inhibition = [A_0_ − (A_1_ − A_2_)]/A_0_ × 100%

where A_0_ represents the absorbance of the DPPH control, A_1_ is the absorbance of the extract–DPPH mixture, and A_2_ is the absorbance of the extract solution without DPPH. Where A_0_ refers to the absorbance of the control solution containing DPPH alone, A_1_ is the absorbance measured for the mixture of the test sample with DPPH, and A_2_ represents the absorbance of the test sample without DPPH.

### 2.4. Biological Study

#### 2.4.1. Ethics and Animal Care

Ethical approval for the study was obtained from the College of Pharmacy Ethics Committee (Reference No. REC/UG/2024/06). All animal procedures were conducted in accordance with the ethical standards of the National Society for Medical Research and the National Institutes of Health Guide for the Care and Use of Laboratory Animals [24].

Female Wistar rats (N = 42, 160–250 g) were procured from the Animal House Facility, College of Pharmacy, Dubai Medical University, Dubai, UAE. Animals were housed in standard ventilated cages under controlled environmental conditions, including a 12 h light/12 h dark cycle and an ambient temperature of 23 ± 2 °C, with free access to a standard pellet diet and water ad libitum. All animals were acclimatized for seven days prior to the commencement of experimental procedures.

#### 2.4.2. Study Design

##### Experimental Design and Treatment Groups

Following an acclimatization period, forty-two female Wistar rats (160–250 g) were randomly allocated into seven experimental groups (n = 6 per group). The experimental groups are as follows:

Group I—Normal untreated rats;
Group II—Diabetic control rats;
Group III—Non-diabetic rats treated with *Salacia reticulata* extract (500 mg/kg/day);
Group IV—Non-diabetic rats treated with *Caralluma tuberculata* extract (500 mg/kg/day);
Group V—Diabetic rats treated with *Salacia reticulata* extract (500 mg/kg/day);
Group VI—Diabetic rats treated with *Caralluma tuberculata* extract (500 mg/kg/day);
Group VII—Diabetic rats treated with metformin (500 mg/kg/day).

Following confirmation of hyperglycemia, treatment groups received the respective plant extracts or metformin at 500 mg/kg/day, suspended in 1% carboxymethylcellulose (CMC), and administered orally by intragastric gavage for 8 consecutive days. The 8-day treatment duration was selected to evaluate the early antihyperglycemic and antioxidant effects of the plant extracts, as reported in previous studies [25].

The extracts’ selected doses of 500 mg/kg/day were chosen based on previously reported toxicological studies demonstrating that the LD_50_ for *Salacia reticulata* is 400 mg/kg/day or higher in Sprague-Dawley rats [26] and *Caralluma tuberculata* is greater than 3 g/kg body weight in mice [27]. No adverse effects, signs of toxicity, or mortality were observed in the animals during the present study.

##### Induction of Experimental Diabetes

**Figure 1:**
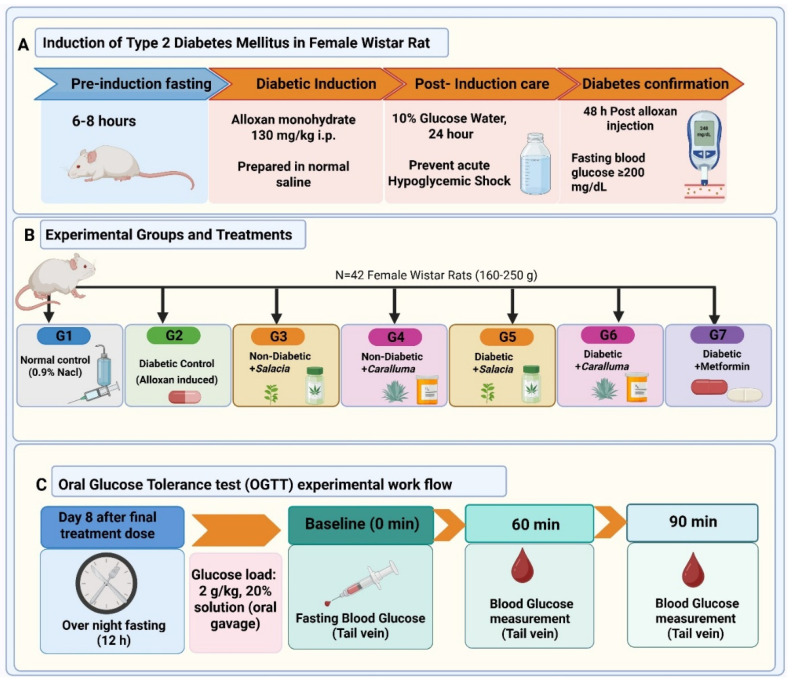
**Schematic representation of the experimental design and oral glucose tolerance test (OGTT) protocol.** (**A**) Alloxan-induced diabetic model in female Wistar rats following 6–8 h fasting by intraperitoneal administration of alloxan monohydrate (130 mg/kg), with post-induction glucose supplementation and confirmation of hyperglycemia (≥200 mg/dL) 48 h later. (**B**) Allocation of animals (n = 6 per group) into control, diabetic control, non-diabetic extract-treated, and diabetic treatment groups receiving *S. reticulata*, *C. tuberculata*, or metformin (500 mg/kg/day) for eight consecutive days. (**C**) OGTT workflow performed on Day 8 after the final treatment dose, including overnight fasting (12 h), oral glucose administration (2 g/kg, 20% solution), and blood glucose measurements at 0, 60, and 90 min.

As shown in Figure 1, rats were fasted for 6–8 h with free access to water prior to diabetes induction. Diabetes was induced by intraperitoneal injection of alloxan monohydrate (130 mg/kg body weight), freshly prepared in sterile normal saline (0.9% NaCl). Alloxan is a well-established diabetogenic agent that selectively damages pancreatic β-cells, leading to hyperglycemia. To prevent acute hypoglycemic shock following alloxan administration, animals were provided with 10% glucose solution in drinking water ad libitum for 24 h after injection [28].

After 48 h of alloxan administration, fasting blood glucose levels were measured using a digital glucometer (Accu-Chek^®^, Roche Diagnostics, Mannheim, Germany) via tail vein puncture. Rats exhibiting fasting blood glucose levels ≥ 200 mg/dL were considered diabetic and included in the study [27]. Metformin was selected as the reference group because it is the first-line treatment for diabetes and one of the most widely accepted antidiabetic agents globally [4]. The pharmacological dose of metformin (500 mg/kg/day) has been extensively used in alloxan-induced diabetic rats [4,7].

##### Blood Glucose Monitoring and Oral Glucose Tolerance Test (OGTT)

Blood glucose levels were monitored to evaluate the hypoglycemic effects of the treatments. Fasting blood glucose was measured 48 h after alloxan administration to confirm hyperglycemia (baseline), and again on Day 3 of treatment. In addition, on the first day of intervention, post-treatment glucose levels were assessed at 60 and 90 min following oral administration of the respective treatments.

On Day 8, an oral glucose tolerance test (OGTT) was performed in the diabetic treatment groups. After an overnight fast (12 h), rats were orally administered a 20% glucose solution (2 g/kg body weight) via intragastric gavage. Blood glucose levels were recorded at 0 (baseline), 60, and 90 min post-glucose load using a digital glucometer (Accu-Chek^®^, Roche Diagnostics, Germany). Glucose response profiles were used to evaluate treatment effects on glucose tolerance.

### 2.5. Tissue and Serum Collection

On the 8th day of treatment, animals were euthanized under sevoflurane anesthesia in accordance with institutional ethical guidelines. Blood samples were collected, followed by excision of the liver and kidney. The tissues were gently rinsed with ice-cold phosphate-buffered saline (PBS) to remove residual blood and immediately fixed in 10% neutral-buffered formalin for histopathological evaluation. The blood was collected aseptically by cardiac puncture into a sterile collection tube without anticoagulant and allowed to clot at room temperature for 30 min. Blood samples were centrifuged at 3000 rpm for 15 min at 4 °C, and the serum was separated, aliquoted into labeled microcentrifuge tubes, and stored at −80 °C until analysis. Samples exhibiting visible hemolysis were excluded.

#### 2.5.1. Histological Analysis

Tissues were fixed in 10% buffered formalin for a minimum of 48 h, dehydrated through graded ethanol (50%, 70%, 90%, 95%, and 100%), cleared in xylene, and embedded in paraffin using an automated tissue processor (Lieca 1020) (Leica, Nussloch, Germany). Paraffin blocks were sectioned at 5 µm, and the ribbons were placed on a 37 °C water bath to ensure adequate spreading. Sections were stained with hematoxylin and eosin (H&E) using a Leica Auto-Stainer XL. Slides were mounted in DPX medium and air-dried for 48 h before microscopic evaluation. Images of the stained sections were acquired with an Olympus BX63 microscope equipped with an Olympus DP75 camera (Olympus Corporation, Tokyo, Japan) (5760 × 3600 pixels, 5.86 × 5.86 µm pixel size) using CellSens Entry software (version 1.17). Images were acquired with 40× (numerical aperture 0.6; 1920 × 1200 pixels; 232.54 nm/pixel resolution in both *X* and *Y* axes) objective lenses.

Histopathological alterations were evaluated using a semi-quantitative scoring system by a blind observer to minimize observational bias. Tissue injury parameters, including inflammatory cell infiltration, cellular degeneration, vacuolation, and architectural disruption, were graded on a scale of 0–3, where 0 = absent, 1 = mild, 2 = moderate, and 3 = severe [29]. Five random microscopic fields per section of each rat per group (n = 5 per group) were evaluated and mean histopathological scores were calculated for each group. Scores for each parameter were averaged per animal, and group data were expressed as mean ± SD.

#### 2.5.2. Analysis of Serum Oxidative Stress and Antioxidant Biomarkers

Serum oxidative stress marker (MDA) and antioxidant biomarkers (SOD1, GSH, and CAT) were quantified using commercially available assay kits (Elabscience, Wuhan, China) according to the manufacturers’ protocols. All samples and standards were analyzed in duplicate, and absorbance was measured using a microplate reader (Tecan, Zurich, Switzerland).

##### Malondialdehyde (MDA) Level Measurements

Lipid peroxidation was evaluated by measuring malondialdehyde (MDA) concentration using an ELISA kit (Elabscience, #E-EL-0060) as per the manufacturer’s instructions. In brief, 50 μL of the serum was added to the ELISA plate wells, and all subsequent steps, i.e., incubations, washings, substrate addition, and stop solution, were performed according to the manufacturer’s protocol. Absorbance was measured at 450 nm using a microplate reader, and MDA levels were calculated from a standard curve and normalized to protein content, expressed as nmol/mg protein.

##### Superoxide Dismutase 1 (SOD1) Level Measurement

Serum levels of superoxide dismutase 1 (SOD1) were quantified using a Rat SOD1 ELISA kit (Elabscience, #E-EL-R1424), and absorbance was measured at 450 nm. SOD1 concentrations were calculated from the standard curve according to the manufacturer’s protocol.

##### Reduced Glutathione (GSH) Level Measurement

Serum reduced glutathione (GSH) concentrations were determined using a competitive GSH ELISA kit (Elabscience, #E-EL-0026), and optical density was measured at 450 nm. GSH levels were calculated using a standard curve generated from known concentrations.

##### Serum Catalase (CAT) Activity

Serum catalase (CAT) activity was measured using a colorimetric catalase activity assay kit (Elabscience,#E-BC-K031-M). This method is based on the enzymatic decomposition of hydrogen peroxide (H_2_O_2_) by catalase. The residual H_2_O_2_ reacts with ammonium molybdate to form a stable yellow complex; the absorbance was measured at 405 nm. Catalase activity was calculated according to the manufacturer’s formula and expressed in units/mg of protein.

### 2.6. Statistical Analysis

Statistical analysis was performed using GraphPad Prism (version 10.2.1; GraphPad Software, Inc., San Diego, CA, USA). The Shapiro–Wilk test was used to assess the normality of the data. Data was analyzed using one-way ANOVA followed by Dunnett’s post hoc test for multiple comparisons. Results are expressed as mean ± SD, and *p* < 0.05 was considered statistically significant.

## 3. Results

### 3.1. Phytochemical Profiling of Plant Extracts

#### 3.1.1. Extraction Yield

Following extraction, the dry yields were 72.00 g for *S. reticulata* and 52.16 g for *C. tuberculata*. The percentage yields calculated based on the initial weight of plant material were 9.00% and 6.52%, respectively. *S. reticulata* exhibited a higher yield (9.0%) compared to *C. tuberculata*.

#### 3.1.2. Total Phenolic and Flavonoid Contents

The highest phenolic and flavonoid contents were recorded in *S. reticulata*, followed by *C. tuberculata* (0.749% and 0.19%; 0.632% and 0.0874%), respectively.

#### 3.1.3. UPLC–MS/MS-Based Metabolite Profiling

**Figure 2:**
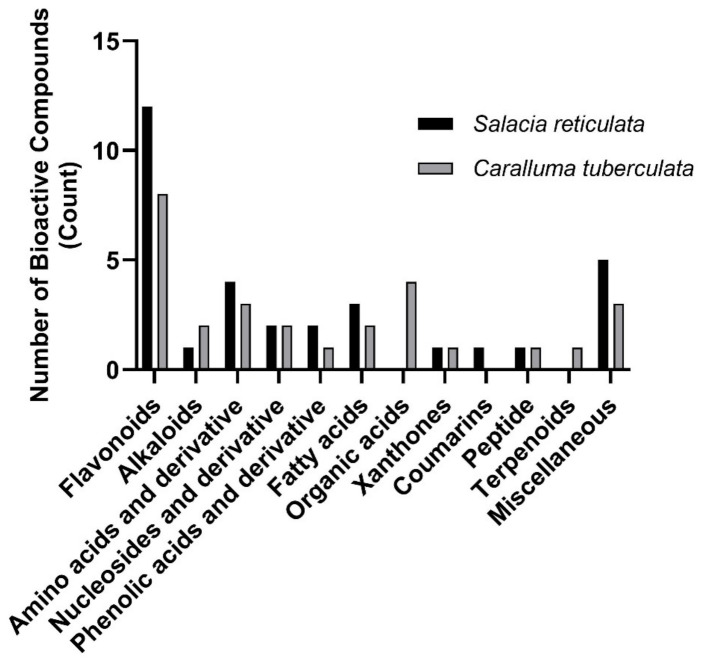
**Comparative distribution of phytochemical classes identified in *S. reticulata* and *C. tuberculata* extracts by UPLC–MS/MS**. Bars represent the number of identified bioactive compounds in each phytochemical class.

UPLC–MS/MS analysis performed in both positive and negative ionization modes identified 33 compounds in *S. reticulata* and 24 compounds in *C. tuberculata* (Table 1 and Table 2; Supplementary Materials). The detected metabolites belonged to diverse phytochemical classes, including flavonoids, phenolic acids, coumarins, alkaloids, amino acids, unsaturated fatty acids, and terpenoids (Figure 2).

Several compounds were common to both plants, including adenosine, luteolin, leupeptin hemisulfate salt, linoleic acid, and elaidic acid. Flavonoids represented a major class in both extracts, with 12 flavonoids identified in *S. reticulata* and 8 in *C. tuberculata*.

Alkaloid profiling revealed caffeine exclusively in *S. reticulata*, whereas trigonelline and o-phenanthroline were detected only in *C. tuberculata*. Notably, *S. reticulata* exhibited a richer polyphenolic profile, including mangiferin, epicatechin, 6,7-dihydroxycoumarin, quercetin and its glycoside (quercetin-4′-glucoside), luteolin-3′,7-di-O-glucoside, cyanidin-3-O-galactoside, cyanidin-3,5-di-O-glucoside, apigenin, and kaempferol-3-O-α-L-arabinoside. Among the identified compounds, mangiferin was the predominant constituent, representing 23.35% of the total peak area. Linoleic acid, an omega-6 fatty acid, and tryptophan, an essential amino acid, were also detected as major compounds in *S. reticulata* extract, accounting for 7.33% and 4.10% of the total peak area, respectively, whereas *C. tuberculata* extract showed a diverse phytochemical profile. Among the detected constituents, kaempferol neohesperidoside and luteolin, both belonging to the flavonoid class, were present as major compounds, representing 9.91% and 3.18% of the total peak area, respectively. In addition, trigonelline, an alkaloid, and linoleic acid, an omega-6 fatty acid, were also detected as major constituents, accounting for 7.59% and 4.96%, respectively.

The comparative phytochemical profile presented in Figure 2 shows clear qualitative and quantitative differences between *Salacia reticulata* and *Caralluma tuberculata* in the number of reported bioactive compounds across different chemical classes. Overall, *S. reticulata* appears richer in several major classes, particularly flavonoids, amino acids and derivatives, nucleosides and derivatives, fatty acids, and miscellaneous compounds. In contrast, *C. tuberculata* shows relatively higher representation in alkaloids and organic acids, while both species display comparable or low representation in xanthones, coumarins, peptides, and terpenoids.

#### 3.1.4. DPPH Radical Scavenging Assay of Plant Extracts

The antioxidant potential of *S. reticulata* and *C. tuberculata* extracts were evaluated using the DPPH radical scavenging method. *S. reticulata* demonstrated strong free radical inhibition with a scavenging activity of 98%. *C. tuberculata* exhibited moderate activity, demonstrating 85.2% effectiveness. Compared with the standard antioxidant, ascorbic acid (which showed 90% inhibition), the order of radical-neutralization effectiveness for the extracts was *Salacia reticulata* > *Caralluma tuberculata*.

#### 3.1.5. Effects of Plant Extracts on Fasting Blood Glucose and Oral Glucose Tolerance

Fasting blood glucose levels remained stable in non-diabetic control groups, and no significant differences were observed before and after treatment with the plants extract alone (Table 3). In contrast, glucose levels significantly increased in the untreated diabetic group after 8 days (560.58 ± 56.90 mg/dL vs. 473.0 ± 132.0 mg/dL; *** *p* < 0.001), indicating progressive hyperglycemia. Treatment with *S. reticulata*, *C. tuberculata*, and metformin significantly reduced fasting blood glucose levels compared with baseline. The percentage reduction in glucose levels was 22.8% for *S. reticulata* (*** *p* < 0.001), 17.6% for *C. tuberculata* (*** *p* < 0.001), and 25.5% for metformin-treated rats (** *p* < 0.01). However, the untreated diabetic group exhibited an 18.5% increase over the same period, and the percentage (%) reduction was calculated as % reduction = (Before − After) × 100.

#### 3.1.6. Oral Glucose Tolerance Test (OGTT)

The oral glucose tolerance test (OGTT) performed on Day 8 showed differences in postprandial glucose responses among the treatment groups (Table 4). The DM + *S. reticulata* group showed the lowest fasting glucose level (108 ± 8.56 mg/dL), followed by a moderate increase at 60 min (136 ± 11.23 mg/dL) and a slight reduction at 90 min (134 ± 9.87 mg/dL). The DM + *C. tuberculata* group showed relatively stable glucose levels, increasing from 122 ± 21.96 mg/dL at baseline to 123 ± 47.08 mg/dL at 60 min and 130 ± 37.54 mg/dL at 90 min.

In contrast, the metformin-treated group showed higher fasting glucose levels (180 ± 36.06 mg/dL) and the greatest increase after glucose administration, reaching 236 ± 0.71 mg/dL at 60 min. However, this group also showed the largest reduction between 60 and 90 min (−22 mg/dL), compared with smaller changes in the *S. reticulata* (−2 mg/dL) and *C. tuberculata* (+7 mg/dL) groups. These findings suggest relatively faster glucose clearance in the metformin-treated group despite poorer baseline glycemic control. Overall, *S. reticulata* showed better fasting glucose stabilization, while metformin demonstrated comparatively improved postprandial glucose clearance. Area under the glucose–time curve (AUC_0–90_) was DM + *S. reticulata* 11,340, DM + *C. tuberculata* 11,498, and metformin 19,530 mg·min/dL, confirming overall lower glucose burden with plant extract treatments.

Thus, *S. reticulata* and *C. tuberculata* appear to offer relative benefits in baseline control and early glucose handling, respectively, whereas metformin appears to promote glucose clearance.

### 3.2. Effect of Plant Extracts on Serum Levels of Oxidant and Antioxidant Biomarkers

**Figure 3:**
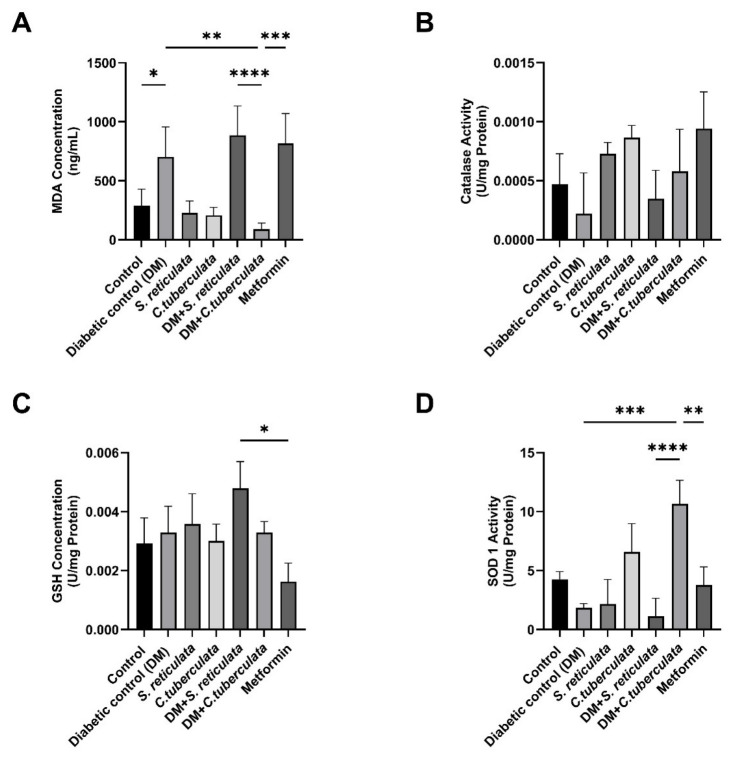
**Serum oxidant and antioxidant biomarker levels in control and treated diabetic rats.** Panels show (**A**) MDA concentration, (**B**) CAT activity, (**C**) GSH concentration, and (**D**) SOD1 activity following 8 days of treatment. Data are expressed as mean ± SD (n = 3–6). * *p* < 0.05; ** *p* < 0.01; *** *p* < 0.001; **** *p* < 0.0001.

Serum oxidative stress markers, lipid peroxidation marker malondialdehyde (MDA), and antioxidant enzyme activities (SOD1 and catalase) were evaluated to assess systemic redox status in alloxan-induced diabetic rats and treatment groups (Figure 3).

#### 3.2.1. Serum Malondialdehyde (MDA) Levels

Serum malondialdehyde (MDA) levels were significantly elevated in diabetic rats compared with controls (*p* = 0.03), indicating increased lipid peroxidation (Figure 3A). Treatment with *S. reticulata* or metformin did not significantly alter MDA levels relative to untreated diabetic rats. In contrast, diabetic rats treated with *C. tuberculata* showed significantly lower MDA levels than the diabetic control group (*p* = 0.002).

#### 3.2.2. Serum Catalase (CAT) Activity

Catalase (CAT) activity was reduced in diabetic rats compared with controls (Figure 3B). Treatment with *S. reticulata* and *C. tuberculata* did not significantly restore CAT activity, whereas the metformin-treated group moderately restored CAT activity relative to untreated diabetic animals, although metformin showed a trend toward increasing CAT activity.

#### 3.2.3. Serum Reduced Glutathione (GSH) Activity

No significant difference in serum reduced glutathione (GSH) levels was observed between diabetic and control groups (Figure 3C). Similarly, treatment with *S. reticulata* or *C. tuberculata* did not significantly modify GSH levels. Metformin-treated rats showed a non-significant decreasing trend compared to DM (*p* = 0.31).

#### 3.2.4. Serum Superoxide Dismutase 1 (SOD1) Activity

Serum SOD1 activity was significantly reduced in diabetic rats compared with controls (Figure 3D). Treatment with *S. reticulata* did not significantly affect SOD1 activity relative to the diabetic group. However, *C. tuberculata* significantly increased SOD1 activity compared with untreated diabetic rats (*p* < 0.0001). Metformin treatment produced a moderate, non-significant increase compared with DM. Antioxidant analysis demonstrated selective restoration of oxidative stress parameters following treatments. SOD1 activity showed better improvement than GSH and CAT levels, suggesting differential responsiveness of antioxidant defense pathways under acute alloxan-induced oxidative stress.

### 3.3. Histopathological Evaluation

**Figure 4:**
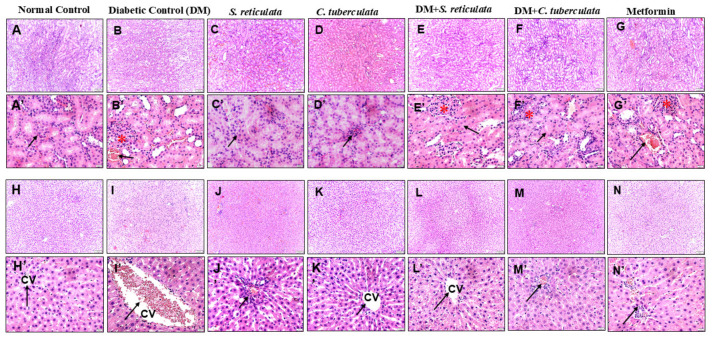
**Histopathological changes in kidney and liver tissues following treatment with *S. reticulata*, *C. tuberculata*, and metformin (H&E staining, 10× and 40× magnification). Kidney:** (**A**,**A′**) The control group showed normal renal architecture with intact glomeruli and tubular structures. (**B**,**B′**) The diabetic group exhibited glomerular distortion, tubular epithelial degeneration, vascular congestion, and inflammatory cell infiltration. (**C**,**C′**–**D**,**D′**) Plant extract-treated groups showed glomerular congestion and interstitial accumulation of inflammatory cells. (**E**,**E′**–**F**,**F′**) *Salacia* and *Caralluma* (500 mg/kg body weight)-treated diabetic rats demonstrating restoration of glomerular and tubular structures respectively. (**G**,**G′**) The metformin-treated group showed largely preserved glomeruli with mild tubular inflammation. **Liver**: (**H**,**H′**) Control group showing normal hepatocyte architecture, organized sinusoidal arrays, and intact central vein (CV). (**I**,**I′**) The diabetic group exhibited perivascular inflammatory infiltration and mild vacuolar degeneration of hepatocytes. (**J**,**J′**–**K**,**K′**) Plant extract-treated groups showed central vein congestion, reduced inflammatory infiltration, and improved hepatocyte organization. (**L**,**L′**) *Salacia*-treated diabetic rats displayed preserved hepatic architecture and sinusoidal arrangement. (**M**,**M’**–**N**,**N′**) The *Caralluma*- and metformin-treated diabetic groups showed minimal inflammatory infiltration and improved hepatocyte integrity, respectively. **Black arrows** indicate areas of inflammatory cell infiltration and degenerative cellular changes in the kidney. Red asterisks (*) denote regions of structural distortion, including glomerular/tubular damage. **CV** denotes the central vein in hepatocyte architecture.

Histopathological analyses of the kidney and liver tissues were performed using hematoxylin and eosin (H&E) staining to characterize diabetes-associated morphological changes and treatment effects (Figure 4). Semi-quantitative histopathological scoring showed significantly higher renal injury scores in the diabetic control group than in normal controls. Treatment with *S. reticulata*, *C. tuberculata*, and metformin significantly reduced tissue injury scores, indicating attenuation of diabetes-induced histopathological damage (Table 5).

Histological examination of kidney sections (renal cortex) (Panels A–G): Renal sections from control rats displayed normal architecture, with well-defined glomeruli and preserved tubular structures (A,A′). On the other hand, diabetic rats exhibited glomerular hypertrophy, tubular epithelial degeneration, interstitial inflammation, vascular congestion, and tubular dilation, consistent with early nephropathic alterations (B,B′).

Non-diabetic rats treated with *S. reticulata* or *C. tuberculata* exhibited preserved renal architecture, with intact glomeruli and normal tubular morphology, comparable to that of control animals (C,C′–D,D′). Treatment attenuated diabetes-induced renal damage. *S. reticulata* produced partial improvement in glomerular and tubular morphology, whereas *C. tuberculata* and metformin treatments demonstrated more pronounced restoration, with improved tubular organization and reduced glomerular distortion (E,E′,G,G′).

Liver histology (Panels H–N) from control animals showed normal hepatic architecture, characterized by organized hepatic cords radiating from the central vein, polygonal hepatocytes with centrally located nuclei, and regular sinusoidal spaces (H,H′). Diabetic rats exhibited disruption of hepatic cord arrangement, hepatocellular vacuolation, sinusoidal dilation, and inflammatory cell infiltration, indicating diabetes-associated hepatic injury (I,I′).

Liver sections from non-diabetic rats treated with plant extracts exhibited preserved hepatic architecture, characterized by well-organized hepatic cords radiating from the central vein. Hepatocytes appeared polygonal with centrally located nuclei, and sinusoidal spaces were regular and intact. No evidence of hepatocellular vacuolation, inflammatory cell infiltration, or structural distortion was observed, indicating that extract administration did not induce detectable hepatic alterations under non-diabetic conditions (J,J′–K,K′). Treatment with plant extracts resulted in structural improvement. *S. reticulata* partially restored hepatic morphology with reduced inflammatory changes (L,L′), whereas *C. tuberculata* and metformin treatments showed more evident normalization of hepatocyte structure and sinusoidal organization (M,M′–N,N′).

## 4. Discussion

Diabetes mellitus remains a growing global health concern, characterized by chronic hyperglycemia, and associated with metabolic, vascular, hepatic, and renal complications [2,9]. Although metformin remains a first-line therapy for glycemic control, its long-term use is associated with gastrointestinal intolerance and limited efficacy in addressing oxidative stress and organ damage [4,5,6]. Therefore, exploring plant-derived therapeutic agents with dual antihyperglycemic and antioxidant potential is of considerable interest. The present study evaluated the aantihyperglycemicand antioxidant effects of *S. reticulata* and *C. tuberculata* in an alloxan-induced diabetic rat model. Standardization of the plants extract showed that *S. reticulata* demonstrated higher total phenolic (0.749%) and flavonoid (0.19%) contents than *C. tuberculata* (0.634% and 0.0874%, respectively), which may explain its stronger antioxidant activity (98% vs. 85.2% DPPH inhibition).

Both extracts significantly reduced fasting blood glucose levels after 8 days of treatment, with *S. reticulata* achieving approximately 25.5% and *C. tuberculata* 12.3%, while metformin showed a comparable effect (~27.4%). The glucose-lowering effects observed in alloxan-induced diabetes may involve multiple mechanisms. Alloxan selectively damages pancreatic β-cells through ROS-mediated injury, reduced intestinal glucose absorption, and impaired peripheral glucose utilization [28].

Regarding the bioactive compounds of the plant extracts, UPLC–MS/MS analysis performed in both positive and negative ionization modes identified 33 compounds in *S. reticulata* and 24 compounds in *C. Tuberculata.* The detected metabolites belonged to diverse phytochemical classes, including flavonoids, phenolic acids, coumarins, alkaloids, amino acids, unsaturated fatty acids, and terpenoids.

Several compounds were common to both plants, including adenosine, luteolin, leupeptin hemisulfate salt, linoleic acid, and elaidic acid. Flavonoids represented a major class in both extracts, with 12 flavonoids identified in *S. reticulata* and eight in *C. tuberculata*.

Among the identified compounds in *S. reticulata*, mangiferin was the predominant constituent representing 23.35% of the total peak area. The antihyperglycemic activity of *S. reticulata* may be attributed to the mangiferin, a natural xanthone compound, which fights diabetes through a multifaceted approach. It combats insulin resistance, lowers post-meal blood sugar by inhibiting carbohydrate-digesting enzymes, stimulates pancreatic beta-cell regeneration, and reduces oxidative stress [30]. In addition, linoleic acid, an omega-6 fatty acid, was detected and represented 7.33% in *S. reticulata* extract, and it was previously mentioned that it had an antidiabetic effect by improving insulin sensitivity, regulating glucose metabolism, and protecting insulin-producing cells [31]. Tryptophan, an essential amino acid, was also detected as a major compound in *S. reticulata* extract, accounting for 4.10% of the total peak area, and it showed promising potential as an adjunctive treatment for diabetes. Studies indicate that it can help lower blood glucose, suppress intestinal glucose absorption, and improve glycemic control [32]. The observed antihyperglycemic activity of the plant may be partly attributed to the possible synergistic effects of mangiferin, linoleic acid and tryptophan, whereas *C. tuberculata* extract showed a diverse phytochemical profile. Among the detected constituents, kaempferol neohesperidoside and luteolin, both belonging to the flavonoid class, were present as major compounds, representing 9.91% and 3.18% of the total peak area, respectively. In addition, trigonelline, an alkaloid, and linoleic acid, an omega-6 fatty acid, were also detected as major constituents, accounting for 7.59% and 4.96%, respectively.

These findings indicate that both plants possess antihyperglycemic potential in the diabetic model employed in this study. This is in agreement with previous reports describing the glucose-lowering effects of these extracts, likely mediated through improved glucose homeostasis and enhanced insulin signaling [11,33]. In addition, plant-derived bioactive compounds have been shown to preserve pancreatic function and improve metabolic regulation in experimental diabetes models [34,35,36,37]. The OGTT findings further supported the differential effects of the treatment on glucose regulation. *S. reticulata* exhibited more efficient fasting glucose stabilization, whereas metformin showed comparatively faster glucose reduction after peak glucose elevation, suggesting improved postprandial glucose clearance kinetics. In contrast, *C. tuberculata* showed a relatively stable and controlled glycemic response throughout the test period. These differences may reflect distinct mechanisms involving intestinal glucose absorption, insulin sensitivity, and peripheral glucose utilization. Previous studies have reported that *S. reticulata* improves glucose homeostasis and enhances insulin sensitivity following carbohydrate challenge [7], while *C. tuberculata* has been associated with reduced intestinal glucose absorption and improved peripheral utilization in diabetic models.

Oxidative stress is a central pathogenic mechanism in alloxan-induced diabetes, where pancreatic β-cell damage is mediated by reactive oxygen species (ROS) generated through redox cycling of alloxan [38,39]. Previous studies have demonstrated that protection against alloxan-induced β-cell toxicity is achieved by suppressing ROS generation and preserving cellular integrity [40]. Consistent with these findings, diabetic rats in the present study exhibited significantly higher serum MDA levels, indicating increased lipid peroxidation. Treatment with *C. tuberculata* significantly reduced MDA levels and selectively improved SOD1 activity, suggesting partial attenuation of oxidative imbalance. In contrast, *S. reticulata* and metformin did not significantly reduce MDA levels despite lowering fasting blood glucose, indicating that their antihyperglycemic effects may not be directly linked to reduced lipid peroxidation.

Catalase activity remained suppressed in plant extracts-treated groups, with only partial improvement in the metformin group, while GSH levels remain unchanged. Mechanistically, the combined reduction in MDA levels and restoration of SOD1 activity suggest that *C. tuberculata* may limit ROS accumulation at an early stage, thereby preventing propagation of lipid peroxidation and cellular damage. This interpretation aligns with the established ROS-dependent toxicity of alloxan and supports a redox-mediated contribution to metabolic improvement [40]. The selective restoration of SOD1 without parallel recovery of CAT or GSH may reflect the upstream positioning of SOD1 in the ROS cascade: SOD1 converts superoxide (O_2_^−^) to H_2_O_2_, which is subsequently neutralized by CAT and GSH peroxidase. The SOD1 restoration by *C. tuberculata* suggests that its bioactive constituent, flavonoids, primarily attenuate superoxide generation at the mitochondrial level, reducing the overall oxidative load before downstream cascades are engaged. The persistent suppression of CAT suggests that alloxan-mediated β-cell injury had already depleted H_2_O_2_-clearing capacity beyond the 8-day duration, while the more abundant superoxide scavenging still reduced downstream lipid peroxidation (evidenced by reduced MDA). This partial restoration is consistent with reports in polyphenol-supplemented diabetic models and indicates that SOD1 normalization alone is sufficient to attenuate oxidative injury without complete multi-enzyme restoration [31,32]. The lower antidiabetic activity of the *C. reticulata*, despite its marked effect on oxidative stress markers, may be explained by the nature of its detected metabolites. The extract contains several antioxidant and cytoprotective constituents, including kaempferol neohesperidoside and luteolin, both belonging to the flavonoid class, which are present as major compounds, representing 9.91% and 3.18% of the total peak area, respectively [34]. These compounds are well known for their ability to reduce oxidative stress through radical scavenging, inhibition of lipid peroxidation, and enhancement of endogenous antioxidant defenses such as SOD-, CAT-, GSH-, GPx-, HO-1-, and Nrf2-related pathways. Therefore, their presence supports the observed improvement in oxidative stress biomarkers [34,35,36].

Together, these findings indicate selective and marker-specific modulation of oxidative stress. Thus, *C. tuberculata* showed the clearest antioxidant-related response, mainly through reduced lipid peroxidation and improved SOD1 activity, but the data do not confirm broad antioxidant activity, β-cell protection, or a specific molecular mechanism.

Comparing the antihyperglycemic and antioxidant effects of both plants extracts, a differential response pattern was evident. *S. reticulata* demonstrated stronger glucose-lowering efficacy but limited effects on oxidative stress markers. In contrast, *C. tuberculata* exhibited pronounced antioxidant activity, with significant reduction in lipid peroxidation and restoration of SOD1 activity, despite a comparatively moderate reduction in blood glucose. The present findings also showed mechanistic differences compared with metformin, which partially restored CAT activity, modestly increased SOD1 levels, and did not significantly reduce MDA levels. This may reflect AMPK-mediated antioxidant effects of metformin [2].

Histopathological observations further supported the biochemical findings by demonstrating substantial tissue injury in diabetic control animals, characterized by tubular/hepatocellular degeneration, congestion, vacuolation, and architectural disruption in both extract-treated groups. Notably, *C. tuberculata* demonstrated moderate tissue recovery, consistent with its antioxidant effect. Similar improvements in tissue histomorphology have been reported for polyphenol-rich plant extracts’ oxidative damage in diabetic models [41]. Treatment with *S. reticulata*, *C. tuberculata*, and metformin significantly reduced overall injury scores, indicating partial tissue recovery and amelioration of diabetes-induced pathological damage. Among the treatment groups, metformin demonstrated the lowest total injury scores, whereas *S. reticulata* showed comparatively greater tissue protection than *C. tuberculata*. These findings suggest that the observed antioxidant and antihyperglycemic effects were accompanied by structural preservation of renal and hepatic tissues under alloxan-induced oxidative stress conditions.

The present study has some limitations that should be considered when interpreting the findings. These include the absence of direct mechanistic and functional assessments. As such, future studies incorporating longer treatment periods, insulin, HOMA-IR and β-cell evaluation, GLUT2/GLUT4 expression, organ-function markers, and isolated-compound validation are needed to clarify the encompassing mechanisms underlying the observed effects.

## 5. Conclusions

The novelty of this study lies in the differential redox–metabolic interplay underlying the antidiabetic effects of *S. reticulata* and *C. tuberculata* extracts in an alloxan-induced diabetic rat model. Both extracts improved fasting glucose levels, modulated oxidative stress markers, and attenuated hepatic and renal structural alterations. Specifically, *C. tuberculata* appears to primarily target oxidative damage by enhancing antioxidant defenses, whereas *S. reticulata* exerts stronger effects on glucose regulation. Importantly, *S. reticulata* demonstrated glucose-lowering efficacy comparable to that of metformin, while *C. tuberculata* demonstrated superior antioxidant restoration, suggesting a dual-functional therapeutic profile. Within the 8-day acute experimental period, both extracts showed short-term antihyperglycemic activity and were accompanied by selective changes in oxidative stress markers and partial attenuation of diabetes-associated histopathological alterations. These preliminary findings support further mechanistic and longer-term studies required to clarify their therapeutic relevance. These findings emphasize the potential advantage of plant-based therapies in simultaneously targeting multiple pathogenic mechanisms of diabetes, including oxidative stress, metabolic dysregulation, and tissue injury. Long-term studies using optimized formulations are needed to further evaluate the sustained efficacy, safety, and mechanistic effects of these extracts, particularly on insulin signaling and glucose metabolism pathways. Such investigations will be important for determining their translational potential as complementary therapeutic candidates for diabetes management.

## Figures and Tables

**Table 1:** Phytochemical profile of *S. reticulata* extract by UPLC–MS/MS.

Serial No.	Compound Name	RT (Min)	% *	Mol. Formula	Mass (*m*/*z*)	Adduct Ion
1	(3R)-4,4-Dimethyl-2-oxotetrahydro-3-furanyl beta-D-glucopyranoside	1.119617	1.66	C12H20O8	315.1108	[M + H]^+^
2	N,N-Dimethylglycine	1.192117	1.03	C4H9NO2	104.1067	[M + H]^+^
3	L-Tryptophan	1.216617	4.10	C11H12N2O2	205.0682	[M + H]^+^
4	D-(+)-Trehalose	1.240283	1.31	C12H22O11	341.1095	[M − H]^−^
5	Glycine-Betaine	1.24095	1.37	C5H11NO2	118.0858	[M + H]^+^
6	Adenosine	1.644967	0.14	C10H13N5O4	268.1037	[M + H]^+^
7	3,4-Dihydroxymandelate	1.796783	0.16	C8H8O5	182.9969	[M − H]^−^
8	Salicylic acid	5.850883	0.90	C7H6O3	137.0242	[M − H]^−^
9	Creatinine	5.852016	0.37	C4H7N3O	114.0907	[M + H]^+^
10	2′-Deoxyinosine 5′-monophosphate	6.207517	0.16	C10H13N4O7P	331.048	[M − H]^−^
11	Mangiferin	6.207517	23.35	C19H18O11	421.0771	[M − H]^−^
12	Caffeine	6.383033	0.15	C8H10N4O2	195.0878	[M + H]^+^
13	Epicatechin	6.50235	0.08	C15H14O6	289.0713	[M − H]^−^
14	[5-(4-hydroxy-3,5-dimethoxyphenyl)-6,7-bis(hydroxymethyl)-1,3-dimethoxy-5,6,7,8-tetrahydronaphthalen-2-yl]oxy]-6-(hydroxymethyl)oxane-3,4,5-triol	6.626367		C28H38O13	600.2659	[M + H]^+^
15	3-Buten-2-one, 4-[4-(beta-D-glucopyranosyloxy)-2-hydroxy-2,6,6-trimethylcyclohexylidene]-	6.721033	0.67 0.06	C19H30O8	409.1822	[M + H]^+^
16	6,7-Dihydroxycoumarin	7.296183	0.06	C9H6O4	177.0194	[M − H]^−^
17	Luteolin-3′, 7-di-O-glucoside	7.357666	0.033	C27H30O16	609.1459	[M − H]^−^
18	2-Phenylethyl beta-D-glucopyranoside	7.5277	0.06	C14H20O6	307.1162	[M + H]^+^
19	Apigenin 8-C-glucoside	7.5013	0.019	C21H20O10	431.0988	[M − H]^−^
20	Delphinidin-3-O-beta-glucopyranoside	7.661283	0.22	C21H21O12	463.0885	[M − H]^−^
21	Cyanidin-3, 5-di-O-glucoside	7.711216	0.05	C27H31O16	611.1638	[M + H]^+^
22	Cyanidin-3-O-galactoside	8.214434	0.17	C21H21O11	447.0933	[M − H]^−^
23	Quercetin-4′-glucoside	8.420083	0.22	C21H20O12	463.0876	[M − H]^−^
24	Kaempferol-3-O-alpha-L-arabinoside	9.26825	0.13	C20H18O10	417.0833	[M − H]^−^
25	Luteolin	10.18647	0.18	C15H10O6	285.0409	[M − H]^−^
26	Quercetin	10.26698	0.05	C15H10O7	301.0354	[M − H]^−^
27	Leupeptin hemisulfate salt	11.2081	0.07	C20H38N6O4	427.2682	[M + H]^+^
28	Apigenin	11.24807	0.05	C15H10O5	269.047	[M − H]^−^
29	3′-Methoxy-4′,5,7-Trihydroxyflavonol	11.70542	0.21	C16H12O7	315.0515	[M − H]^−^
30	Palmitoleic acid	21.69667	0.11	C16H30O2	253.216	[M − H]^−^
31	L-Cystine	13.62247	0.04	C6H12N2O4S2	241.0294	[M + H]^+^
32	Linoleic acid	22.15155	7.33	C18H32O2	279.2332	[M − H]^−^
33	Elaidic acid	23.34465	0.73	C18H34O2	281.2484	[M − H]^−^

* Relative percentages of the identified compounds in the plant extracts were calculated based on the total peak area in the chromatogram [20,21].

**Table 2:** Phytochemical profile of *C. reticulata* extract by UPLC–MS/MS.

Serial Number	Compound Name	RT (Min)	% *	Mol. Formula	Mass (*m*/*z*)	Adduct Ion
1	Trigonelline	1.259817	7.59	C7H7NO2	138.0547	[M + H]^+^
2	Mucate	1.346383	0.51	C6H10O8	209.0296	[M − H]^−^
3	Galactarate	1.346383	0.51	C6H10O8	209.0296	[M − H]^−^
4	(S)-MALATE	1.384050	11.82	C4H6O5	133.014	[M − H]^−^
5	L-5-Oxoproline	1.435883	0.17	C5H7NO3	128.0346	[M − H]^−^
6	L-pipecolic acid	1.591533	0.40	C6H11NO2	128.0354	[M − H]^−^
7	Adenosine	1.653333	0.94	C10H13N5O4	268.1042	[M + H]^+^
8	D-Alloisoleucine	1.756983	0.34	C6H13NO2	132.102	[M + H]^+^
9	2′-Deoxyadenosine	3.91385	0.42	C10H13N5O3	252.109	[M + H]^+^
10	5-O-Caffeoylquinic acid methyl ester	4.033367	0.56	C17H20O9	367.1248	[M − H]^−^
11	1,3,6,7-tetrahydroxy-2-[(2S,3R,4R,5S,6R)-3,4,5-trihydroxy-6-(hydroxymethyl) oxan-2-yl]xanthen-9-one	6.125967	2.47	C19H18O11	421.0779	[M − H]^−^
12	Kaempferol-7-neohesperidoside	8.049867	9.91	C27H30O15	593.1498	[M − H]^−^
13	Rhoifolin	8.5731	1.00	C27H30O14	579.1712	[M + H]^+^
14	Cyanidin-3-glucoside	8.671267	0.12	C21H21O11	449.1078	[M + H]^+^
15	Cynaroside	8.376350	0.17	C21H20O11	447.0917	[M − H]^−^
16	(2R,3R,4S,5S,6R)-2-octoxy-6-[[(2S,3R,4S,5R)-3,4,5-trihydroxyoxan-2-yl]oxymethyl]oxane-3,4,5-triol	9.332517	0.45	C19H36O10	469.2282	[M − H]^−^
17	Luteolin	10.183770	3.18	C15H10O6	285.0404	[M − H]^−^
18	Leupeptin hemisulfate salt	11.22347	0.22	C20H38N6O4	427.2658	[M + H]^+^
19	Peonidin	11.526030	2.43	C16H13O6	299.0555	[M − H]^−^
20	Cucurbitacin I	17.093250	0.14	C30H42O7	513.3038	[M − H]^−^
21	o-Phenanthroline	17.58423	0.19	C12H8N2	181.1217	[M + H]^+^
22	2,5-dihydroxy-3-undecylcyclohexa-2,5-diene-1,4-dione	18.333050	0.12	C17H26O4	293.2113	[M − H]^−^
23	Linoleic acid	22.31898	4.96	C18H32O2	279.2331	[M − H]^−^
24	Elaidic acid	23.23907	1.65	C18H34O2	281.2486	[M − H]^−^

* Relative percentages of the identified compounds in the plant extracts were calculated based on the total peak area in the chromatogram [20,21].

**Table 3:** Effect of *S. reticulata*, *C. tuberculata,* and metformin on fasting blood glucose levels in alloxan-induced diabetic rats.

Group	Before (mg/dL)	After (mg/dL)	Reduction (%)
Control group	116.3 + 13.4	130 + 13.5	+11.8
Diabetic control (DM)	473.0 + 132.0	560.58 + 56.9 ***	+18.5
*S. reticulata* only	139.8 ± 16.5	113.5 ± 11.5	−18.8
*C. tuberculata* only	131.8 ± 29.7	108.17 ± 19.41	−17.6
DM + *S. reticulata*	279 ± 79.65	207.83 ± 81.75 ***	−25.5
DM + *C. tuberculata*	172.5 ± 44.2	151.25 ± 42.04 ***	−12.3
Metformin	196.0 ± 76.5	142.17 ± 26.01 **	−27.4

** *p* < 0.01; *** *p* < 0.001.

**Table 4:** Oral glucose tolerance test (OGTT) in treated diabetic rats.

Group	0 min (Fasting)	60 min	90 min
DM + *S. reticulate*	108.00 ± 8.56	136.00 ± 11.23	134.00 ± 9.87
DM + *C. tuberculate*	122.00 ± 21.96	123.00 ± 47.08	130.00 ± 37.54
Metformin	180.00 ± 36.06	236.00 ± 0.71	214.00 ± 13.44

Blood glucose levels (mg/dL) at baseline (0), 60, and 90 min.

**Table 5:** Effects of *S. reticulata*, *C. tuberculata*, and metformin on histomorphometric indices of kidney and liver in alloxan-induced diabetic rats.

**Kidney**					
**Group**	**Tubular degeneration**	**Inflammatory infiltration**	**Architectural damage**	**Total injury score**	
Normal control	0.10 ± 0.09	0.02 ± 0.04	0.00 ± 0.00	0.18 ± 0.13	
Diabetic control	2.65 ± 0.19	2.62 ± 0.12	2.53 ± 0.08	7.8± 0.31	
*S. reticulate*	0.18 ± 0.08	0.06 ± 0.05	0.03 ± 0.05	0.26 ± 0.10	
*C. tuberculate*	0.20 ± 0.09	0.07 ± 0.05	0.05 ± 0.05	0.32 ± 0.11	
DM + *S. reticulata*	1.22 ± 0.08	1.12 ± 0.08	1.04 ± 0.05	4.44 ± 0.15	
DM + *C. tuberculata*	1.43 ± 0.06	1.30 ± 0.10	1.27 ± 0.06	5.27 ± 0.15	
Metformin	1.00 ± 0.08	0.90 ± 0.08	0.87 ± 0.05	3.70 ± 0.1	
**Liver**					
**Group**	**Hepatocyte degeneration**	**Sinusoidal congestion**	**Vacuolation**	**Central vein congestion**	**Total injury score**
Normal control	0.08 ± 0.08	0.05 ± 0.05	0.00 ± 0.00	0.02 ± 0.04	0.17 ± 0.08
Diabetic control	2.60 ± 0.14	2.62 ± 0.15	2.45 ± 0.10	2.28 ± 0.08	12.50 ± 0.51
*S. reticulate*	0.10 ± 0.09	0.02 ± 0.04	0.03 ± 0.05	0.02 ± 0.04	0.17 ± 0.11
*C. tuberculate*	0.13 ± 0.08	0.03 ± 0.05	0.07 ± 0.05	0.03 ± 0.05	0.27 ± 0.12
DM + *S. reticulata*	1.18 ± 0.08	1.12 ± 0.08	1.05 ± 0.05	1.05 ± 0.05	5.48 ± 0.21
DM + *C. tuberculata*	1.42 ± 0.08	1.28 ± 0.08	1.25 ± 0.05	1.15 ± 0.05	6.37 ± 0.10
Metformin	1.02 ± 0.08	0.95 ± 0.05	0.85 ± 0.05	0.90 ± 0.06	4.63 ± 0.21

## Data Availability

Data are contained within the article and in the Supplementary Materials.

## Supplementary Materials

The following supporting information can be downloaded at: https://www.mdpi.com/article/10.3390/pharmaceutics18070785/s1, Figure S1: LC-Mass analysis using positive mode TIC, (**A**): *Salacia reticulata*; (**B**): *Caralluma tuberculata*; Figure S2: LC-Mass analysis using positive mode BPC, (**A**): *Salacia reticulata*; (**B**): *Caralluma tuberculata*; Figure S3: LC-Mass analysis using Negative mode TIC, (**A**): *Salacia reticulata*; (**B**): *Caralluma tuberculata*; Figure S4: LC-Mass analysis using negative mode BPC, (**A**): *Salacia reticulata*; (**B**): *Caralluma tuberculata*.

## Abbreviations

The following abbreviations are used in this manuscript:

AlCl_3_	Aluminum chloride
a.u.	Directory of open access journals
CAT	Catalase
CMC	Carboxymethylcellulose
Da	Dalton
DM	Diabetes mellitus
DPPH	2,2-Diphenyl-1-picrylhydrazyl
DPX	Distrene Plasticizer Xylene
GAE	Gallic acid equivalents
g	Gram
GS1	Gas 1
GSH	Reduced glutathione
H&E	Hematoxylin and eosin
h	Hour
HSS	High-strength silica
HPLC	High-performance liquid chromatography
LC	Liquid chromatography
LC-MS	Liquid chromatography–mass spectrometry
LD_50_	Lethal dose, 50%
MDA	Malondialdehyde
mg	Milligram
mL	Milliliter
min	Minutes
µg/mL	Microgram per milliliter
µm	Micrometer
MS1	First mass analyzer scan
MS2	Second mass analyzer scan
Na_2_CO_3_	Sodium carbonate
NaNO_2_	Sodium nitrite
NaOH	Sodium hydroxide
OGTT	Oral glucose tolerance test
SOD1	Superoxide dismutase 1
TFC	Total flavonoid content
TOF-MS	Time-of-flight mass spectrometry
TPC	Total phenolic content
T2DM	Type 2 diabetes mellitus
UPLC-MS/MS	Ultra-performance liquid chromatography–tandem mass spectrometry
UV-Vis	Ultraviolet–visible
V	Voltage
WHO	World Health Organization
