# Supplementary material for: Antihyperglycemic and Antioxidant Effects of Salacia reticulata and Caralluma tuberculata in Alloxan-Induced Diabetic Female Rats
Source: Pharmaceutics. 2026 Jun 26;18(7):785. doi: 10.3390/pharmaceutics18070785 (PMC13414625; doi:10.3390/pharmaceutics18070785)
Supplement: Supplementary file 1 [file pharmaceutics-18-00785-s001.zip › pharmaceutics-4285871-supplementary.pdf]

Supplementary Material

Traditional Medicine vs. Modern Pharmacotherapy: Efficacious Herbal Antidiabetic Agents Compared to Metformin in Female Rats

Positive -MODE – TIC

A.

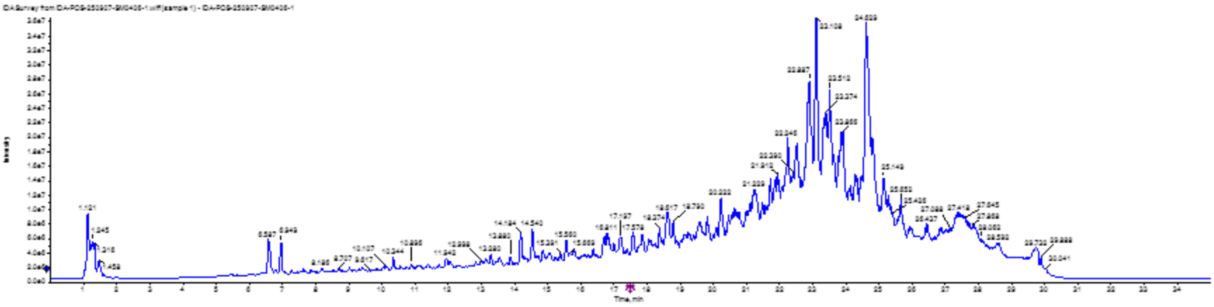

B.

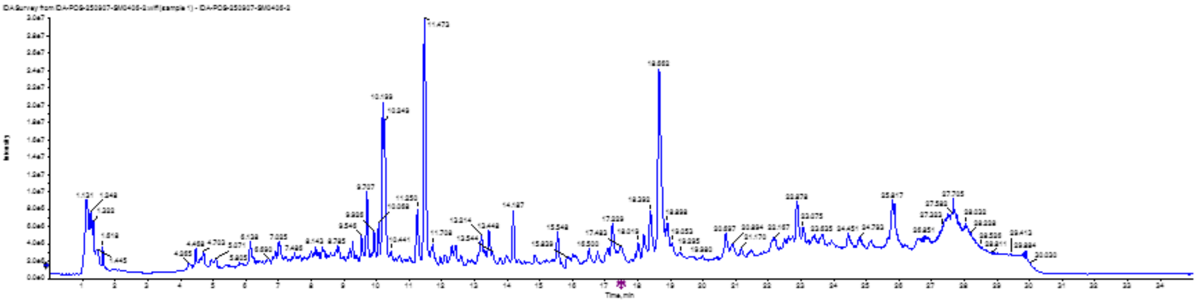

Supplementary Figure S1: LC-Mass analysis using positive mode TIC, **A**: *Salacia reticulata;* **B**: *Caralluma tuberculata*

Positive -MODE – BPC

A.

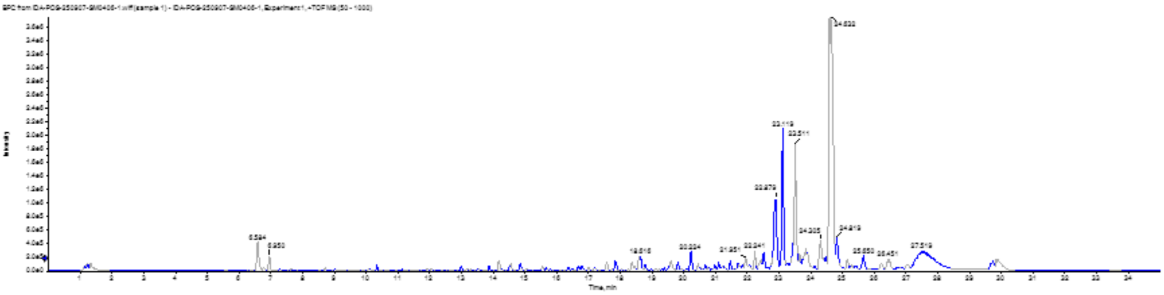

B.

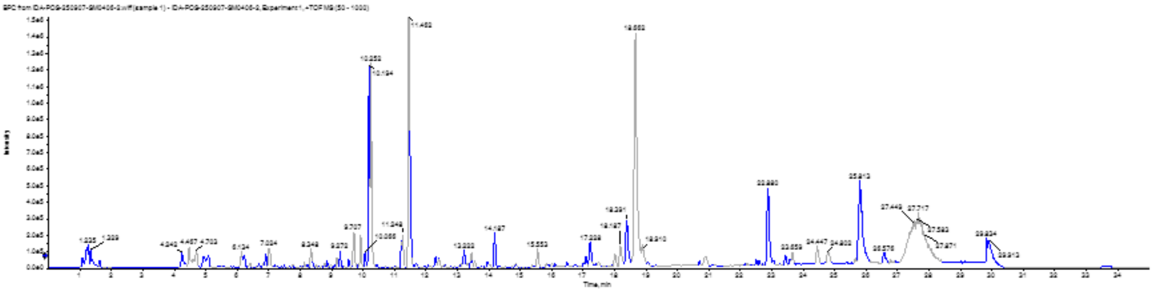

Supplementary Figure S2: LC-Mass analysis using positive mode BPC , **A**: *Salacia reticulata;* **B***: Caralluma tuberculata*

Dubai Health - Internal Use

Negative -MODE – TIC

A.

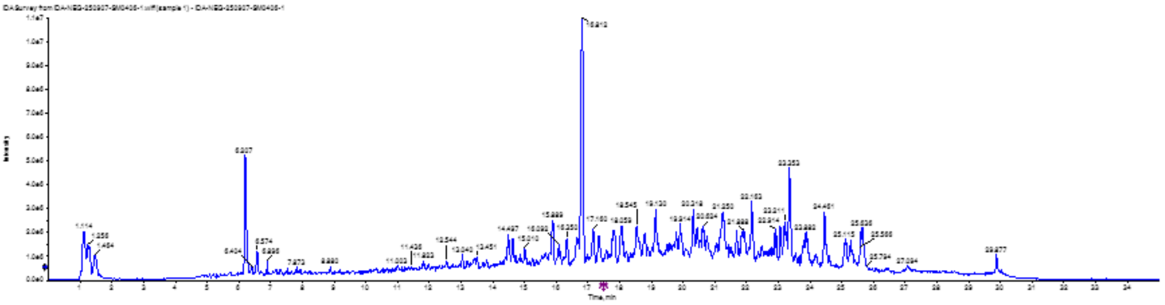

B.

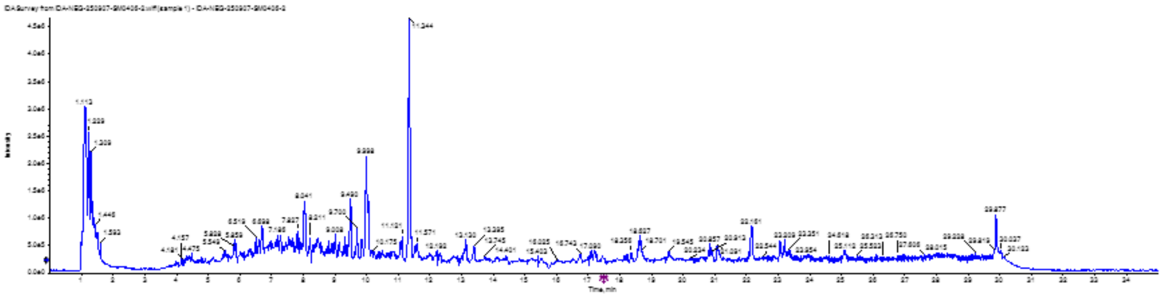

.

Supplementary Figure S3: LC-Mass analysis using Negative mode TIC, **A**: *Salacia reticulata;* **B***: Caralluma tuberculata*

Negative -MODE - BPC

A.

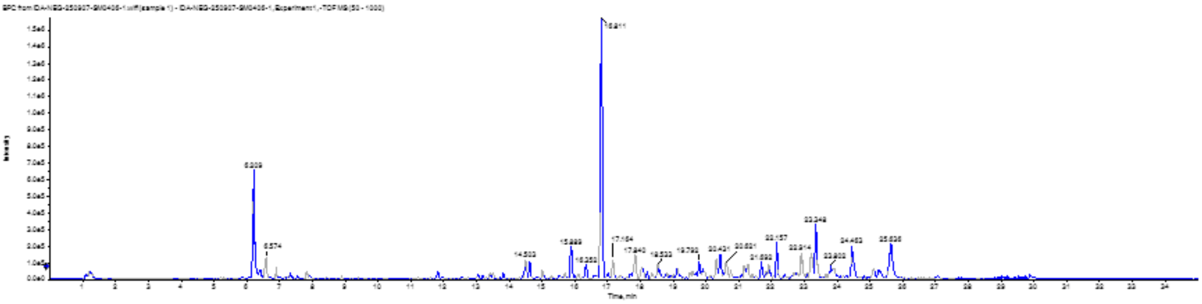

B.

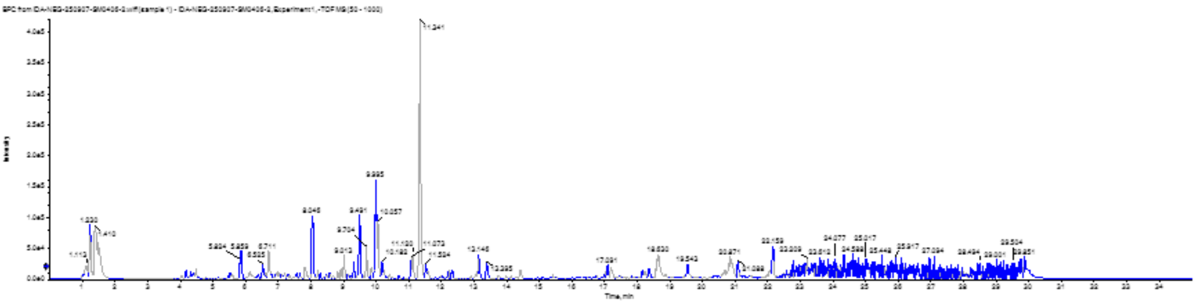

Supplementary Figure S4: LC-Mass analysis using negative mode BPC, **A**: *Salacia reticulata;* **B**: *Caralluma tuberculata*

Dubai Health - Internal Use
